# Prevalence and Determinants of Routine Childhood Vaccination Hesitancy in Makkah, Saudi Arabia

**DOI:** 10.7759/cureus.63270

**Published:** 2024-06-27

**Authors:** Mrnan Sulaimani, Siham Al-Mehmadi, Moayed Sulaimani, Salman Alsubhi

**Affiliations:** 1 Preventive Medicine and Public Health, Ministry of Health, Makkah, SAU; 2 College of Dentistry, Umm Al-Qura University, Makkah, SAU

**Keywords:** parents, pacv, children, hesitancy, vaccine

## Abstract

Background: Vaccines are among the most important inventions of the last century; they contribute significantly to preventing infectious diseases. In 2019, the World Health Organization (WHO) recognized vaccine hesitancy as one of the top ten threats. This study aimed to estimate the prevalence and determinants of routine childhood vaccine hesitancy among parents in Makkah City in 2023 using the Parent Attitude about Childhood Vaccine (PACV) survey.

Methods: A cross-sectional study was conducted from October to December 2023 among parents of children aged six years or younger who attended primary healthcare centers (PHCC) in Makkah City using a stratified sampling technique. Data were collected using an electronic self-administered questionnaire, and the Arabic PACV Cronbach's alpha was 0.79.

Results: A total of 246 parents participated in the study. Over half of the participants were males (56.5%), and the mean age was 36 ± 7.2. Parents who scored 50% or more were considered hesitant. The study identified approximately 3% of parents as hesitant. The only significant association toward hesitancy status was age; younger parents were less hesitant than older parents, P-value < 0.006. The other variables, such as gender, educational level, marital status, employment status, household income, number of children, and having a child with chronic disease, were not significantly associated with vaccine hesitation.

Conclusion: Though the overall parental hesitation rate is low, several questions received more hesitant responses than non-hesitant responses. Therefore, we recommend raising awareness through healthcare providers focusing on educating parents and correcting misconceptions about the safety and efficacy of vaccines.

## Introduction

Vaccines are one of the most important inventions of the last century; they contribute significantly to preventing infectious diseases, despite the fact that there is still hesitancy toward vaccines. It is a major challenge for public health, and it was recognized by the World Health Organization (WHO) in 2019 as one of the top ten threats [1]. The vaccine hesitancy definition was recently modified by the WHO in May 2022 into “a motivational state of being conflicted about, or opposed to, getting vaccinated; this includes intentions and willingness.” This new definition separates the intention from the resulting behavior, which may lead to a better understanding of the reasons behind the hesitation [2]. There are multiple studies around the world showing a decline in vaccination uptake due to concerns [3-5]. The threat of vaccine hesitancy in recent years appeared in under-vaccination, which led to an increase in the incidence of vaccine-preventable disease outbreaks such as measles, poliomyelitis, and pertussis [6-8].

To begin with, a cross-sectional study by an infectious diseases international research initiative conducted in 16 countries found that the overall vaccine hesitancy rate among parents is 13.7% [9]. Furthermore, an overview of vaccine hesitancy stated that the factors that influence the decision to vaccinate are complex and have many aspects, including emotional, cultural, social, cognitive, and political factors [10]. In addition, a systematic review published in September 2022 by Obohwemu et al., which aimed to understand the reasons behind vaccine hesitancy, showed that mistrust of health officials and suspicion about vaccine safety and effectiveness were the most mentioned barriers [11].

After the pandemic of COVID-19, there has been a noticeable increase in interest in vaccine hesitancy research. Regarding Saudi Arabia, there are few studies conducted exploring the prevalence and determinants of vaccine hesitancy of COVID-19 among parents, but even fewer studies investigating hesitancy of routine or other childhood vaccinations such as influenza and human papillomavirus. A study conducted in Riyadh in 2018 by Alsubaie et al. found that 20% of the parents were hesitant, 36% of children of hesitant parents were partially vaccinated for their age, and the most common cause of vaccine hesitancy was concerns about vaccine safety, followed by fear of side effects and mistrust in the effectiveness of vaccines [12]. Another study in Aseer in 2020 by Alqahtani et al. showed that around 20% of participating parents were hesitant and did not fully adhere to the routine vaccination schedule [13]. In addition, a study conducted in Hail in 2021 found an association between vaccine hesitancy and age; the prevalence of hesitancy among parents who were less than 40 years old was 21.4%, compared to 35% in parents who were over 40 years old [14].

Therefore, this study was designed to estimate the prevalence and determinants of routine childhood vaccine hesitancy among parents in Makkah City in 2023 using the Parent Attitude about Childhood Vaccine (PACV) survey [15], which is a self-administered survey to investigate parental hesitancy and includes 15 questions covering three domains: behavior, safety and efficacy, and general attitude and trust. The tool has been validated to identify hesitancy in parents and has been widely used and translated into many languages. Finally, given the importance of this topic to the clinical practice of public health, it is surprising that no studies have explored routine childhood vaccination hesitancy in Makkah city; hence, this study was aimed to answer the question and help fill the gap in vaccine hesitancy research in Saudi Arabia.

## Materials and methods

Study setting and population

This cross-sectional study was conducted from October to December 2023 among parents who attended primary healthcare centers (PHCC) in Makkah City, using a stratified sampling technique. Primary healthcare centers in Makkah are divided into four sectors (East, West, North, and South), and one PHCC was selected randomly from each sector based on a list provided by a simple random generator. This study was carried out on parents of children aged six years or younger who showed up at outpatient clinics at selected PHCC; we excluded the parents with children aged more than six years, immunocompromised children, and who came only for vaccination to avoid selection bias. The sample size was calculated using EPI-INFO 7 software [Centers for Disease Control and Prevention (CDC), Atlanta, Georgia, USA], estimating 20% prevalence according to the literature [12,13] with a 95% confidence interval and 5% marginal error. The resulting sample was 246 participants, and 62 parents who met the eligibility criteria from each PHCC were approached.

Data collection and tool

We collected data using an electronic, self-administered questionnaire. We approached parents who were in the waiting rooms and asked them to participate. Parents who were eligible were joined after getting their consent. Filling out the survey was optional, and no incentives were used. The questionnaire contains 2 parts; the first part covers socio-demographic data such as age, gender, nationality, marital status, employment status, educational level, household income, number of children, and age of the youngest child. It also covers two more questions regarding the vaccination status of the children. The second part involves PACV tools that were developed to evaluate parents’ vaccine hesitancy and have been used in many studies. It contains 15 questions covering three domains: items 1 and 2 cover behavior; items 3 to 6 and 11 to 15 are linked with general attitude and trust; and items 7 to 10 are related to safety and efficacy. The calculation of points was as follows: 2 points for a hesitant response, 1 point for a do not know response, and 0 points for a non-hesitant response. PACV scores range from 0 to 30, and a non-hesitant parent was defined with a score of <15 and a hesitant parent with a value of ≥15. An Arabic version of the tool was developed by Alsuwaidi et al. Content validity was tested by the investigators using forward and backward translation; reliability was tested, and Cronbach's alpha was 0.79 [16].

Ethical consideration

The research proposal was approved by the institutional review board of Security Force Hospital in Makkah, registered at the National Bio-Medical Ethics Committee, King Abdulaziz City for Science and Technology, on 71711436 (Registration No.: HAP-0 2-K-052) with the approval number (0595-240523). Permission was obtained from the director of each PHCC before starting data collection. Informed consent was obtained from all participants after explaining the study’s aim with the assurance that their data would be anonymous and used for research purposes only.

Statistical analysis

For data analysis, we used Statistical Package for the Social Sciences (SPSS) software version 23 (IBM Corp., Armonk, NY). Frequencies and percentages were used to summarize categorical variables, while numerical variables were presented as mean and standard deviations (SD). A chi-square test was employed to assess the association between the hesitancy status and each of the independent variables. Significant associations were considered for tests with a P-value less than 0.05.

## Results

Parents’ basic characteristics

A total of 246 parents participated in the study; their sociodemographic characteristics are shown in Table 1. Over half of the participants were males (56.5%), the mean age was 36 ± 7.2, and the majority of parents were aged between 31 and 40 years old (53.7%). Around 60% of them were employed, and a bachelor’s degree or higher education was achieved by most of the parents (56%).

**Table 1:** Characteristic of participants (N = 246).

Socio-demographic characteristics	Number	Percent
Age group		
20–30	63	25.6
31–40	132	53.7
41–50	41	16.7
More than 50	10	4
Gender		
Male	139	56.5
Female	107	43.5
Nationality		
Saudi	277	92.3
Non-Saudi	19	7.7
Educational level		
School degree	69	28
Diploma	39	15.9
Bachelor	120	48.8
Higher degree	18	7.3
Employment status		
Student	4	1.6
Unemployed	87	35.4
Employed	147	59.8
Retired	8	3.2
Marital status		
Married	240	97.6
Divorced	4	1.6
Widowed	2	0.8
Household income		
Less than 5000 SR	42	17.1
5000 to 10,000 SR	109	44.3
More than 10,000 to 15,000 SR	67	27.2
More than 15,000 SR	28	11.4
Number of children		
1	55	22.4
2	61	24.8
3	47	19.1
4	45	18.3
More than 4	38	15.4
Is your child having a chronic disease?		
No	215	87.4
Yes	31	12.6
Did your children get the seasonal influenza vaccine?		
No	157	63.8
Yes	89	36.2
Did your children get the COVID vaccine?		
No	187	76
Yes	59	24

Parents’ attitude toward childhood vaccines

**Figure 1:**
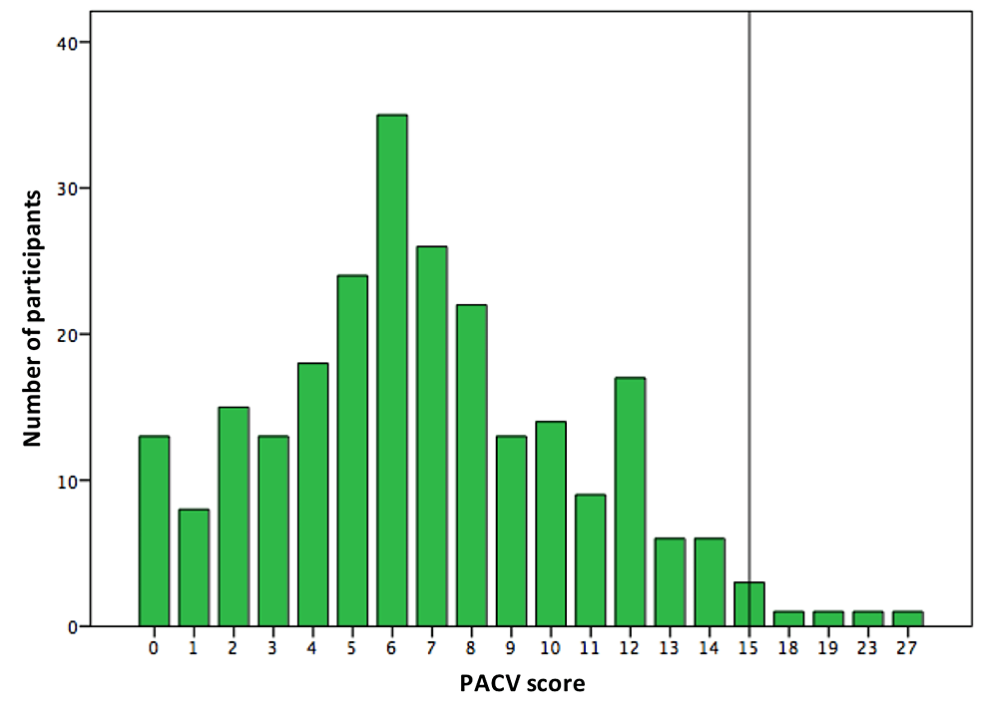
Participant Parent Attitudes about Childhood Vaccines (PACV) scores, with reference line at score 15.

The median PACV score was 6, with an interquartile range of (4-9). Parents who scored 50% or more were considered hesitant. Although in question 12, around 13% of parents expressed themselves as hesitant, only 2.8% of parents were identified as being hesitant. The reference line is shown in Figure 1. Table 2 showed that 23% of participants delayed their children’s shots, and 14 parents decided not to vaccinate their children for reasons other than illness or allergy. Moreover, around 20.7% of respondents disagree or are unsure about the severity of diseases prevented by vaccines, and 61.4% of parents believed that getting immunity by having the disease was better than vaccination. However, regarding the trust domain, 93% of parents trust the information they received about vaccination, 90% trust their pediatricians, and 80% feel able to discuss their concerns with their child’s doctor. Though the final score showed most parents are non-hesitant, 45% of non-hesitant parents thought their children were getting more vaccinations than necessary, 67% of them thought it was better for the children to get fewer vaccines at the same visit, 54% were concerned that their child might have a serious side effect from a shot, 43.5% were concerned that the shot may not be safe, and 49% were hesitant or unsure if a vaccine would prevent the disease.

**Table 2:** Responses to individual PACV statements by 246 participants. PACV: Parent Attitude about Childhood Vaccine.

No.	item	Response	Number	Percent
1	Have you ever delayed having your child get a shot for reasons other than illness or allergy?	Yes	48	19.5
		No	190	77.2
		Don’t know	8	3.3
2	Have you ever decided not to have your child get a shot for reasons other than illness or allergy?	Yes	10	4.1
		No	232	94.3
		Don’t know	4	1.6
3	How sure are you that following the recommended shot schedule is a good idea for your child? From 0 (not at all sure) to 10 (completely sure).	0–5	26	10.5
		6–7	8	3.3
		8–10	212	86.2
4	Children get more shots than are good for them.	Agree	45	18.3
		Disagree	136	55.3
		Unsure	65	26.4
5	I believe that many of the illnesses that shots prevent are severe.	Agree	195	79.3
		Disagree	18	7.3
		Unsure	33	13.4
6	It is better for my child to develop immunity by getting sick than to get a shot.	Agree	111	45.1
		Disagree	95	38.6
		Unsure	40	16.3
7	It is better for children to get fewer vaccines at the same time.	Agree	96	39
		Disagree	78	31.7
		Unsure	72	29.3
8	How concerned are you that your child might have a serious side effect from a shot?	Concerned	110	44.7
		Not concerned	109	44.3
		Unsure	27	11
9	How concerned are you that anyone of the childhood shots might not be safe?	Concerned	64	26
		Not concerned	135	54.9
		Unsure	47	19.1
10	How concerned are you that a shot might not prevent the disease?	Concerned	55	22.4
		Not concerned	122	49.6
		Unsure	69	28
11	If you had another infant today, would you want him/her to get all the recommended shots?	Yes	227	92.3
		No	12	4.9
		Don’t know	7	2.8
12	Overall, how hesitant about childhood shots would you consider yourself to be?	Hesitant	22	8.9
		Not hesitant	214	87
		unsure	10	4.1
13	I trust the information I receive about shots.	Agree	229	93.1
		Disagree	9	3.7
		Unsure	8	3.3
14	I am able to openly discuss my concerns about shots with my child’s doctor.	Agree	201	81.7
		Disagree	25	10.2
		Unsure	20	8.1
15	All things considered; how much do you trust your child’s doctor? From 0 (no trust at all) to 10 (completely trust).	0–5	25	10.1
		6–7	20	8.2
		8–10	201	81.7

Association between sociodemographic characteristics and hesitancy

Table 3 demonstrates the only significant association toward hesitancy status was age; younger parents were less hesitant than older parents, P-value < 0.006. The other variables, such as gender, educational level, marital status, employment status, household income, number of children, and having a child with chronic disease, were not significantly associated with vaccine hesitation.

**Table 3:** Association between vaccine hesitancy and sociodemographic characteristics using chi square test.

Sociodemographic characteristics	Hesitant	Non hesitant	P value
Age group			
20–30	1	62	0.006
31–40	2	130	
41–50	2	39	
More than 50	2	8	
Gender			
Male	6	133	0.114
Female	1	106	
Nationality			
Saudi	7	220	0.437
Non-Saudi	0	19	
Educational level			
School degree	2	67	0.488
Diploma	0	39	
Bachelor	5	115	
Higher degree	0	18	
Employment status			
Student	4	0	0.103
Unemployed	87	0	
Employed	141	6	
Retired	7	1	
Marital status			
Married	7	233	0.914
Divorced	0	4	
Widowed	0	2	
Household income			
Less than 5000 SR	2	40	0.351
5000 to 10,000 SR	2	107	
More than 10,000 to 15,000 SR	1	66	
More than 15,000 SR	2	26	
Number of children			
1	0	55	0.139
2	2	59	
3	0	47	
4	2	43	
More than 4	3	35	
Is your child having a chronic disease?			
No	5	210	0.196
Yes	2	29	
Did your children get the seasonal influenza vaccine?			
No	6	151	0.221
Yes	1	88	
Did your children get the COVID vaccine?			
No	4	183	0.235
Yes	3	56	

## Discussion

This study aimed to estimate the prevalence and determinants of routine childhood vaccine hesitancy among parents in Makkah city using the PACV tool, which was developed in 2011 and has been used in many studies since then to successfully identify hesitancy in parents [15].

The overall hesitancy was around 3%, which is lower than stated in the systematic review by the Infectious Diseases International Research Initiative, which covers 16 countries, and the overall hesitancy rate was around 14% [9]. Also, lower than several studies conducted in the United States of America [17], Malaysia [18], the United Arab Emirates [16], Ireland [19], and Italy [20], which used the PACV survey and found the rate to be (26%), (11.6%), (12%), (6.7%), and (7.7%), respectively. The difference between these findings may be due to the different characteristics of the population studied and their cultures.

Saudi Arabia is a large country. There are some studies conducted about parental hesitancy toward childhood vaccinations across different cities, and the overall rate was 20% in Riyadh [12], 20% in Aseer [13], 10.6% in Taif [21], and the rate of non-compliance was 7% in Madinah [22] and 38% in Najran [23]. A possible explanation for the variation between results could be related to the different tools used for conducting and measuring the outcome.

Moreover, about 50 parents delayed their children’s vaccines for reasons other than illness or allergy, but only 14 parents refused to get their children vaccinated; the specific reason is unknown. However, the study done by Alyami et al. [23] revealed that the main reasons for delaying vaccination are forgetting the appointment and the shortage of vaccines. For future research, we suggest using follow-up space for both questions 1 and 2 to provide reasons that may lead to better understanding.

However, despite the low total rate of hesitancy, the parents’ main concern was the safety and efficacy of the vaccine, and this was revealed in questions that recorded hesitant responses more than non-hesitant responses, such as item 7 (68.3%), item 8 (55.7%), item 9 (45.1%), and item 10 (50.4%), followed by item 4 (44.7%) and item 6 (61.4%), which fall under the attitude domain. These findings aligned with several other studies done in Saudi Arabia [12,24], and worldwide [25,26], reporting that the main parental worry about vaccines was safety doubts. Hence, we suggest planning awareness campaigns or formulating health education materials, emphasizing the focus on the safety of vaccines.

Notwithstanding the fear of the safety of vaccines, our study found great trust in healthcare officials and providers, which presented the highest score of questions covering the trust domain. Around 82% completely trust their pediatrician and 93% trust the information received about vaccines. Thus, it is a good sign and reflection of the massive efforts made by the Ministry of Health in Saudi Arabia towards vaccination. This finding is inconsistent with a systematic review by Obohwemu et al., which concluded that mistrust of health officials was one of the main barriers [11]. Trust toward healthcare providers is an important factor in hesitancy. Therefore, it is essential to ensure doctors educate parents about the benefits of getting vaccinated and the harms of avoiding it.

In this present study, the only determinant significantly associated with hesitancy was the age variable, which is coherent with the findings of Alnumair et al. [14] and Altulaihi et al. [27]. Both studies revealed that the prevalence of hesitancy was higher in parents older than 40 compared to younger parents, and the probable justification for these conclusions is the effect of health education that led to increasing the awareness of the population.

In addition, investigating other factors, including educational level or income level, showed no significant association, similar to the results found by Alamri et al. [28]. Moreover, our study showed no relation between hesitancy and material status, unlike the conclusion of Alsuwaidi et al. [16], which found hesitancy significantly correlated with divorced parents. The likely explanation is the low participation rate of divorced or widowed parents.

While this study has many strengths, it also includes a few limitations that should be considered. As with any self-administered questionnaire method, there is a possibility for recall bias. Additionally, the lack of advanced statistical analysis, such as regression analysis, to predict factors was inapplicable because there was no significant association factor in the univariate analysis.

## Conclusions

In conclusion, this study was the first to address routine childhood vaccine hesitancy among parents in Makkah city, and its findings would help in knowing the extent of the issue. Though the overall parental hesitation rate is low, there are several questions that received more hesitant responses than non-hesitant responses. Therefore, we recommend raising awareness through healthcare providers to prevent increasing the hesitation rate, and we emphasize focusing on educating parents and correcting misconceptions about the safety and efficacy of vaccines.
